# Neuromarketing as an Emotional Connection Tool Between Organizations and Audiences in Social Networks. A Theoretical Review

**DOI:** 10.3389/fpsyg.2020.01787

**Published:** 2020-07-21

**Authors:** Natalia Abuín Vences, Jesús Díaz-Campo, Daniel Francisco García Rosales

**Affiliations:** ^1^Department of Applied Communication Sciences, Complutense University of Madrid, Madrid, Spain; ^2^Faculty of Business and Communication, International University of La Rioja, Madrid, Spain

**Keywords:** social media, emotions, neuromarketing, organizations, audiences

## Abstract

Currently, there is an important debate on how social networks have affected relations between organizations and their audiences: originally complementary –since organizations had full control over the messages that they sent to users, who were mere consumers of information–, they are now symmetric –since users produce and disseminate information about organizations on a global scale through social media–. Therefore, one of the main concerns of organizations when investing in social networks is to connect with their target audience, to have virality, greater visibility and scope. Likewise, neuromarketing is gaining significant importance when it comes to predicting user behavior through biometric measurements, so it can be an essential tool for developing content that engages organizations and their audiences. The main objective of this work is to conduct a theoretical review of the main scientific research on the effectiveness of neuromarketing as a tool to improve the emotional connection between organizations and users in social networks. Thus, the scientific literature on the object under study available on the Web Of Science has been extensively reviewed. The results of the analysis of the main researches in this field reveal the importance of neuromarketing: some of them agree that the communicative effectiveness between organizations and audiences in social networks depends more on sociology and psychology than on technology itself. Neuromarketing has also allowed to demonstrate the relevance of the so-called social influence in social networks: users tend to imitate the behaviors of others, under the premise that these actions reflect the appropriate procedure. That is, when a user sees that others in their environment comment or share a post, they tend to replicate that action in order to avoid the fear of being the only one who behaves differently.

## Introduction

Neuroscience is introducing new ways to understand various fields of scientific knowledge, among them, its contributions to understand the operation and effects of advertising on potential consumers must be highlighted. Morin (2011) indicates that the concept comes from the combination of “neuro” and “marketing,” which implies the fusion of two major fields of study (neuroscience and marketing). Neuroscience was developed by Gerald Zaltman, and aims to help marketers understand how the human brain is physiologically affected by advertising and marketing strategies (Lee et al., 2007). It is one of the newer branches of the advertising industry, as it is an emerging interdisciplinary field linking the knowledge of psychology and neuroscience to marketing (Gurgu et al., 2020). This science is in an embryonic state, as marketing professionals are just beginning to unveil the brain circuits involved in finding, choosing and purchasing a product. While many of the studies conducted by neuromarketers are commercial and, as such, do not go through the standards nor the review process imposed by academics, enough evidence has already been published to highlight some neurocognitive principles at play when consumers perceive advertising messages (Morin, 2011).

Ariely and Berns (2010) affirm that marketers are enthusiastic about this new science for two fundamental reasons. Firstly, because they believe that these types of techniques will make it possible to offset costs and benefits. This hope is based on the idea that consumers are not able to expressly articulate their purchasing preferences when explicitly asked, and that their brain possesses hidden information about their true predilections. Such information could be used to influence their purchasing behavior so that the cost of conducting neuroimaging studies would be offset by the benefit of better product design and higher sales. In theory, at least, brain imaging could shed light not only on what people like but also on what they will buy. The second reason is that they hope it will provide an accurate market research method that can be implemented even before a product exists. Nonetheless, Fisher et al. (2010) indicate that neuromarketing raises important professional, ethical and scientific concerns. This new field exemplifies the complicated question of professional ethics applied to academic-business relationships. Furthermore, as it is a new application of neuroscience methods, it presents important considerations for responsibly conducting research and its public understanding.

## Materials and Methods

The objective of this article is to review the most recent investigations that analyze neuromarketing as a tool that connects consumers and organizations through social networks. This review highlights the conceptualization of the relationship between disseminated content on social networks and the effect that these platforms have on users’ emotional responses. In order to contextualize this review, selected articles had to meet the following requirements: they focus on the study of social networks, carry out a research about the possibilities of these platforms for generating emotions and/or analyze the effects they produce on the user. Based on these criteria, the article is structured around three main blocks: a first section, which introduces the concept of neuromarketing and its influence on consumer decision-making; a second section, in which the characteristics and possibilities of social networks as platforms for generating emotions are reviewed; and a third section, focused on the effects they produce on the user:

1. Neuromarketing and consumer decision-making.
2. Social networks and emotions: content, language, tools, and possibilities.
3. Social networks and emotions: user reactions, determining elements, and engagement implications.

This review is descriptive in nature, as it aims to provide an update on the concept of neuromarketing in relation to a constantly evolving medium such as social media.

The first criterion for the selection of articles was their presence in the Web of Science platform since it includes the references of the main scientific publications of any discipline of knowledge, both scientific and technological, humanistic and sociological, since 1945. The second criterion was novelty, as in, that their publication date was as recent as possible. In this sense, it is necessary to highlight that the discipline of neuromarketing is relatively young and, if included to the study of relationships between companies and consumers in social networks, the bibliographic corpus is considerably reduced.

In order to present and synthesize the characteristics of the included studies, the aforementioned eligibility criteria have been followed. A total of 75 articles were selected. The earliest publication date was 2000 and the most recent, 2020.

It is important to highlight that the articles reviewed which were published between 2000 and 2010 mainly refer to the concept of neuromarketing and its application in the analysis of advertisements in conventional mass media.

Table 1 provides detailed data on the publications covered in this study.

**TABLE 1:** Research on neuromarketing and emotions in social networks.

Thematic area	References	Social network studied	Object of study	Geographical area
Business communication	Chmiel et al., 2011	Digg, blogs, and BSS forums	Virtual communities	International
	Cvijikj and Michahelles, 2013	Facebook	100 brands	International
	Dessart, 2017	Facebook	48 Facebook pages and 448 consumers	International
	de Vries et al., 2012	Facebook	11 brands	International
	Goh et al., 2013	Facebook	FFS Retailer	Asia
	Hollebeek and Chen, 2014	Facebook	Apple and Samsung Mobile fan communities	International
	Hudson et al., 2015	Facebook and YouTube	423 users	United States
	Hudson et al., 2016	Social media in general	Approximately 1,000 participants	France, United Kingdom, and United States
	Khan et al., 2016	Facebook	McDonalds, Kentucky Fried Chicken (KFC), Burger King (BK), Subway, and Domino’s	Australia, United Kingdom, and United States
	Kim et al., 2015	Facebook	92 brands	International
	Kim and Yang, 2017	Facebook	10 Fortune companies and 10 companies on Wall Street’s 24/7 list of most hated companies	United States
	Marbach et al., 2016	Facebook	28 users from a Facebook fan community	International
	Moussa, 2019	Twitter	18 worldwide brands	International
	Rout et al., 2018	Twitter	Apple, Google, Microsoft, and Twitter	International
	Schultz, 2017	Facebook	6 retail apparel brands and 7 retail food brands	International
	Swani et al., 2017	Facebook	Fortune 500 companies	International
	Swani and Milne, 2017	Facebook	Fortune 500 companies	International
	Teixeira et al., 2012	Social media in general	58 students	United States
	Vignal Lambret and Barki, 2018	Facebook, Twitter, and YouTube	RATP, SNCF, Samarco, Skol, Air France, and Coca Cola	France and Brazil
	Xu and Wu, 2017	Twitter	253 participantes de Amazon Mechanical Turk	United States
Advertising	Aguirre et al., 2015	Facebook	2 retail brands	Netherlands
	Guixeres et al., 2017	YouTube	8 Super Bowl TV commercials	Europe
	Mañas-Viniegra et al., 2019	Instagram	120 university students	Spain and Portugal
	Muñoz-Leiva et al., 2019	Facebook	Hotel Jardín Tropical (Tenerife, Spain)	Spain
Scientific communications	Gómez-Adorno et al., 2016	Twitter	Author profiles in PAN 2015 and PAN 2016	International
	Hwong et al., 2017	Facebook and Twitter	50 public pages with space science content	International
	Smith and Seitz, 2019	Facebook	744 participants of MTurk	United States
Cultural communications	Chang et al., 2019	Unspecified	4528 PTT portal movie reviews	China
Sports communications	Lee and Kahle, 2016	Twitter	4 MLB teams and Nike, Adidas, Reebok and Under Armor	United States
Personal comunication	Barry et al., 2019	Instagram	100 university students	United States
	Brynielsson et al., 2014	Twitter	Tweets related to Hurricane Sandy	International
	Carrillo et al., 2015	Twitter	Tweets collected from Twitter API	International
	Coviello et al., 2014	Facebook	Facebook users	International
	Fan et al., 2018	Weibo	Weibo users	China
	Ferrara and Yang, 2015	Twitter	3,800 Twitter users	International
	Lee and Hong, 2016	Facebook	420 students	Corea
	Lin and Utz, 2015	Facebook	401 users	Germany and the United States
	Mauri et al., 2011	Facebook	30 students	Italy
	Meshi et al., 2013	Facebook	31 participants	Germany
	Min and Yun, 2019	Social media in general	1,200 users	South Korea
	Nash et al., 2019	Instagram	77 university students	New Zealand
	Nelson-Field et al., 2013	Facebook, Twitter, and blogs	800 shared videos on social media	International
	Ng and Kozlowski, 2018	Social media in general	300 users	Australia and Singapore
	Ranganathan and Tzacheva, 2019	Twitter	200,000 preprocessed tweets from Twitter API	International
	Sion, 2019	Facebook, Twitter, Instagram, WhatsApp, Snapchat, and Pinterest	Data from the Georgia Institute of Technology, Mashable, Pew Research Center, Suggestme, Statista, and Tech Infographics	United States
	Tandoc et al., 2014	Facebook	736 students	United States
	Turel et al., 2018	Facebook	32 participants	United States
	Vermeulen et al., 2018	Facebook, Twitter, Instagram, Snapchat, and Messenger	Messages from 22 teenagers	Belgium
	Wang et al., 2017	Facebook, Twitter, Snapchat, Tumblr, Tinder, Google+, and Whisper	275 selfies and group posts	International
Political communications	Stieglitz and Dang-Xuan, 2013	Twitter	Tweets referring to the main German political parties	Germany

**Source: Own elaboration*.*

## Results

### Neuromarketing as a Tool for Anticipating Consumer Decision-Making

Bault and Rusconi (2020) indicate that, in recent years, knowledge on the neurobiology of choice has increased significantly. Research in the field of decision-making has identified important brain mechanisms that construct a representation of an option’s subjective value based on previous experience, recovered, and compared with that of other options available to choose from. Lim (2018) ensures that neuroscientific methods encapsulate the use of tools and techniques to measure, map, and record brain and neuronal activity during behavior and, in doing so, generate neurological representations of that activity to understand specific responses in the brain and in the nervous system as a result of exposure to a stimulus. These methods, which allow neuroscientists to observe the neural processes that occur during behavior in real time, can be classified into three broad categories: neuroscientific tools and techniques that record neural activity within (electromagnetic and metabolic) and outside the brain, and neuroscientific methods to manipulate neural activity.

Ambler et al. (2000) carried out two small-scale experiments with neuromarketing techniques, in order to determine the effects of emotional and rational advertising on users exposed to it. The authors based their study on the results of previous research that indicated that emotional advertising generated higher levels of recognition and memory than purely cognitive. Their first experiment tested these conclusions with conventional methods before using pharmacological treatments (β blockers), to see if reducing the impact of affection also reduced the difference between remembering and recognizing both types of advertising. The second experiment used Magneto-Encephalography (MEG) to investigate whether there were distinguishable patterns of brain activation in time and space between affective and cognitive advertising. In the preliminary experiment, recall and recognition of affective advertisement were significantly stronger for both the control and placebo groups. Recall decreased in the group which used the drug. The results related to recognition were not definitive. Harris et al. (2019) researched the use of consumer neuroscience to improve and determine the effectiveness of ads related to public health and social causes in digital media. This study showed that action/emotion-based marketing communications that ask people to act, share, promise or challenge tend to be more effective than those based on rationality. Also, none of the highest attention peaks were produced when viewing the brand logos. Besides, Hafez (2019) explains that marketing specialists must develop a positive and favorable brand image in the minds of customers through the development of attractive ads with emotional content. Neuromarketing research has empirically evidenced that most purchasing decisions are made emotionally. Therefore, creating initiatives to build an emotional bond is the main task of experts to improve marketing performance.

Neuromarketing has allowed to analyze how the type of medium in which advertising is inserted impacts the emotional reaction of the viewer. Baraybar-Fernández et al. (2017) carried out a research focused on discovering the relationship between the emotions induced in audiovisual advertising messages and their impact on the subject’s memory. To achieve this, they carried out an experiment with eight audiovisual advertising messages (six representatives of six basic emotions: joy, surprise, anger, disgust, fear and sadness; and two rational ones). On the one hand, they used neuromarketing techniques such as the cardiac electrical activity (ECG) and the electrical activity of the dermis (AED) of the subjects. On the other, a conventional research technique was also used: a questionnaire applied to the subjects who participated in the research. The results showed that, both for the suggested memory of the message transmitted and for the activity of the advertiser, the announcement with the best results was that of sadness, an announcement that was also considered the most attractive by the subjects under study. Accordingly, Vecchiato et al. (2014) carried out an experiment to investigate cognitive and emotional changes in brain activity evaluated by neurophysiological indices while watching television commercials. In particular, they recorded electroencephalogram (EEG), galvanic skin response (GSR), and heart rate (HR) in a group of 28 healthy subjects while watching a series of television commercials that were grouped by category. They performed brain index comparisons to highlight gender differences between categories and scenes of interest from two specific ads. The results show how EEG methodologies, together with measurements of autonomous variables, can be used to obtain hidden information from advertisers that is not otherwise accessible. One of the main findings was to determine that these tools allow analyzing the perception of television advertisements and differentiating their production according to the gender of the target audience.

These techniques have also been effective in analyzing the effects of print advertising. For example, Dos Santos et al. (2019) analyzed how sponsorships functioned in sports posters. The authors’ objective was to examine the influence of congruence (perceived and effective) and the level of visual attention toward sponsors on recall as well as purchase intention in sports sponsorship by applying neurophysiological measures. The experiment used eye tracking techniques with 111 men and 129 women (*n* = 24) with 24 sports posters from three different disciplines (sailing, tennis, and F1), with varying consistency, number of sponsors, and position. The results showed that the recall of the brand is influenced by the number of sponsors present on the poster and by the time of fixation. Likewise, it has been shown that the use of sexual claims in advertisements published in print media does not increase brand recall, compared to those that do not use this type of strategy (Fidelis et al., 2017).

Guixeres et al. (2017) studied whether it was possible to predict the effectiveness of advertisements on digital channels by using neural networks and metrics based on neuroscience (brain response, heart rate variability, and eye tracking). The neurophysiological records of 35 participants were exposed to eight Super Bowl television commercials. Correlations between metrics based on neurophysiology, ad recall, ad *likes*, the audience rating provided by ACE metrix, and the number of YouTube views over a year were investigated. Results suggest a significant correlation between neuroscience metrics, the advertising effectiveness self-report, and the direct number of visits on the YouTube channel. This study is a pioneer in the use of neurophysiological methods to predict advertising success in a digital context. Likewise, some researchers have shown that the electroencephalography (EGG) technique can provide indications about a subject’s interest in watching a video or the possibility of closing and skipping it without seeing it (Libert and Van Hulle, 2019).

In 2011, Kendall Goodrich analyzed the relationship between attention to online advertising, attitude toward the brand, suggested memory, and purchase intention. Thus, attention tracking techniques were used in a controlled online environment. The results of this experiment suggest that attention is positively related to the suggested memory and purchase intention, but negatively related to the attitude toward the brand.

With the advent of web 2.0, neuromarketing is providing interesting data to advertisers on the effectiveness of their advertising on social networks. Muñoz-Leiva et al. (2019) carried out research on travel advertising on social networks and showed that it is more effective when inserted in media with little editorial content such as Facebook or specialized blogs. They also showed that the use of celebrities as a claim in these types of ads captures the attention of potential consumers.

In their study on social cognitive processes and neural systems, Meshi et al. (2015) described the social motives that drive people to use social networks and proposed systems for their use. The use of social networks occurs for two main reasons: connecting with others and managing the impression they leave on others. People try to satisfy their basic social needs on these platforms and adopt behaviors based on social cognition, thinking about the mental states and motivations of other users; self-referential cognition, publishing information about themselves; and social reward processing, social connection suggestion, or reputation enhancement. Meshi et al. (2013) studied the relationship between the way the brain processes earnings specifically relevant to reputation and the degree of use of Facebook. In their study, the authors demonstrate that, when users respond to gains in self-reputation, relative to observing the gains of others, the intensity of users’ involvement with Facebook can be predicted. Turel et al. (2018) research the excessive and compulsive use of social networks in order to understand the brain systems and processes that are involved in addition to these platforms. Symptoms of addiction to social networking sites are manifested in usage behaviors that focus on immediate profits and weighing their misuse with future consequences.

Using neuromarketing for social media analysis enables companies to look past big data and go beyond the socially desired responses, as it brings to light real reactions. Therefore, the effort has a great final reward. However, to be sustainable, since this is a joint effort (companies need the help of consumers for data collection), the communication strategy should focus on showing consumers how they are benefited (Constantinescu et al., 2019).

### Emotions: Content, Language, Tools and Possibilities in Social Networks

User engagement and participation have become central non-transactional concepts in the new era of marketing. The work of Cvijikj and Michahelles (2013) analyzes how the characteristics of the content communicated by a company on Facebook affect user behavior. The authors focused on the type of medium, the type of content, the day and time of publication, the number of likes, comments, shared actions, and duration of interaction on the brand page on this platform. Their results suggests that entertainment content is the most influential, posts with information related to the brand increase the level of engagement through likes and comments, photos are the most attractive type of publication medium, and the amount of comments is higher in posts shared on weekdays. Khan et al. (2016) analyzed the impact of cultural differences on social networks and the commitment, loyalty and brand recommendations of users. According to these authors, videos are an influential element and improve the number of likes, comments, and shares. The number of comments tends to be higher in this type of content and when the brand’s posts stays for a longer time at the top of the page. However, this work shows that CSR-related posts do not improve the number of comments nor the number of times content is shared. de Vries et al. (2012) analyzed possible factors that drive the popularity of brands’ posts on social media. Their study on eleven international brands determined that the position of the post at the top of the brand’s fan page improves its popularity, and that positive comments on a brand’s posts is positively related to the number of likes. Kim et al. (2015) studied the marketing practices implemented on Facebook by the world’s leading brands in order to detect the qualitative factors of the messages most likely to generate a consumer response. Consistent with the studies noted above, the results of this research indicate that images attract more consumer responses than those based solely on text and, on several occasions, tend to receive more responses than video content. According to this study, the content published more frequently on the pages of this social network is oriented toward interaction, something that may be due to the intention of promoting customer-brand relationship in the long term.

The characteristics of the content disseminated on social networks affect the forms of user interaction, but so do the sector and the characteristics of the organization. Schultz (2017) also studied user participation in brand posts, considering their characteristics, duration, number of fans, and industry. This author identified differences in user participation depending on activities and industries. According to the study, environmental variables such as market and target group characteristics affect consumer engagement with brand messages. Therefore, social media strategies must consider market and target group segmentation. Swani and Milne (2017) studied how the Fortune 500 companies’ brand content strategies favor the reach of popularity on Facebook, analyzing the differences between brands of goods and of service. The results of their work show that the use of corporate brands is more popular in service-related posts, while the use of product brands, images, and videos is more popular for product posts. According to these authors, posts related to services generate more comments than those related to goods. Comparing business-to-business (B2B) models and business-to-consumer (B2C) models, Swani et al. (2017) analyzed brand content published on Facebook by Fortune 500 companies in B2B markets compared to B2C models. These authors studied the key factors that influence the popularity of the content of this social network, based on the theory of psychological motivation. Their results indicate that the inclusion of corporate brand names, functional and emotional appeals in messages, the lack of direct calls to purchases or sales, and the inclusion of informative content increases the popularity of B2B messages compared to B2C messages. Shen et al. (2017) researched whether media-based emotions can be used to predict future commodity market returns. These authors provided more evidence on the effects that news and emotions based on social networks have on the commodity market. Hwong et al. (2017) studied the participation of users in science-related messages on Facebook and Twitter. Through supervised learning algorithms, they identified several unique characteristics of space science communications. These authors presented a predictive model to forecast the levels of user participation in posts. Their results indicate that the levels of interaction in the messages related to space science in social networks can be predicted with an accuracy of close to 90% using only content-based features. This study identifies anger and anxiety in messages, linked to pressing global problems such as climate change or disasters due to natural phenomena, the rarity of safe and positive publications related to this field, and the good reception by the public of messages with positive emotions and visual elements as exclusive characteristics of this field.

The identification of feelings and the analysis of the opinions of individuals disseminated on social networks facilitate the understanding of public opinion and the recommendation of content on these platforms for users. In Smith and Seitz’s (2019) paper on correcting neuroscience myths via Facebook, it is evident that readers evaluate articles more positively when they are consistent with pre-existing opinions. However, their study suggests that submitting articles related to correcting those myths immediately after exposure to misinformation may reduce belief in them. However, the research by Vermeulen et al. (2018) on the social exchange of emotions between adolescents on social networks indicates that updates on Facebook, Instagram, and Snapchat are mainly used to share positive emotions, while Twitter and Messenger are used to share negative emotions. The research by Goh et al. (2013) analyzed the interaction between users and administrators on clothing brand pages on Facebook, considering the impact of content created by consumers and sellers. Their results show that participation in brand communities of social networks leads to an increase in purchasing expenses and that the social impact of user-generated content is stronger than the content published by the administrator of these pages to stimulate consumer buying behavior. Kim and Yang (2017) analyzed how the actions of commenting, sharing, and reacting to Facebook posts can be used to improve the ranking of users’ feelings. According to these authors, behaviors such as liking, commenting, and sharing contribute to the classification of feelings and are necessary for calculating feelings polarity. Hong and Cameron’s (2017) results show that, in a crisis situation, users tend to consider the reputation of organizations more positively when they read online comments defending the company, compared to when they only read the news. According to these authors, comments can motivate people to redirect the crisis in a positive direction.

Given the vast content generated on social networks and the increasing amount of information, Chang et al. (2019) proposed a method for analyzing the emotional aspects of the Chinese vocabulary and evaluating the massive comments of movie reviews on social platforms. Their approach improves the effectiveness of recommendation systems, based on machine learning and emotional information. In order to share valuable information at the right time, Lee and Kahle (2016) analyzed the linguistic composition of the content of social networks in sports, specifically the communication of teams and sports equipment companies on Twitter. These authors presented a framework for understanding the choice of certain words in sports communication, their association with social interests, the complexity of thought, and other psychological processes. Also, Ranganathan and Tzacheva (2019) proposed a model for the automatic detection of emotions in Twitter messages. Considering the emotions of the user, their research allows extracting rules of action to provide suggestions with a wide variety of applications in teaching, customer satisfaction, or business improvement models, following the automatic data classification model Support Vector Machine LibLinear, by Fan et al. (2008). Through machine learning, Rout et al. (2018) identified feelings from unstructured data, specifically on Twitter and SMS (messages via mobile phones). These authors evaluate the utility of supervised and unsupervised algorithms for the classification of these feelings. For their analysis, they generated a test lexicon in their corpus and took advantage of Google’s search engine to determine the score of each term using precise mutual information. Carrillo et al. (2015) proposed a tool for the study of semantic structures, dependent on time, based on the social network Twitter. This measure of time-dependent semantic similarity is validated for use in synonyms in cases that do not involve a highly specialized semantic space, such as a given professional field, and allows semantics to be defined using more colloquial language expressions.

Beyond verbal rating systems, Moussa’s (2019) study focused on a non-verbal mechanism: the emoji. This author introduced a new emoji-based metric for monitoring consumer emotions toward brands on social media, associated with the American Customer Satisfaction Index (ACSI). The author suggested that this abbreviated communication mechanism may be more diagnostic than complete statements. Gómez-Adorno et al. (2016) presented a lexical resource to preprocess social network data based on neural networks and also includes systems of non-verbal mechanisms: emoticons. This research on PAN 2015 and PAN 2016 author profiles includes slang word dictionaries, contractions, abbreviations, and emoticons commonly used on social media in English, Spanish, Dutch, and Italian.

The collection of user data and the self-learning of these tools must be carried out without the user perceiving that their privacy is being violated. Aguirre et al. (2015) demonstrated that when companies collect information about users to offer them personalized online advertising, the expected results are not always achieved since it can make consumers feel that their privacy is being violated. Their exploratory field study on Facebook showed sharp falls in the click-through rate when customers realized that their personal information had been collected without their consent. When companies collect user data in an open way, they exhibit higher click intentions in response to personalized ads, as opposed to when companies covertly collect information. The effect reflects the feelings of vulnerability that consumers experience when companies engage in covert information-gathering strategies.

Regarding crisis communication, Vignal Lambret and Barki (2018) analyzed how the emotions of online stakeholders can help companies face a crisis in social networks and, consequently, minimize the threat of reputation. These authors presented a crisis management matrix on social media and emphasized the need for flexible, stakeholder-focused approaches that can influence crisis development and resolution. Xu and Wu (2017) studied the effect of incorporating sympathy through social networks to counteract psychological reactance in crisis communication. The results of their research suggest that using Twitter and expressing sympathy significantly reduces reactance.

Posting images of users on social media can be linked to aspects related to self-esteem or specific themes correlated with narcissism and concerns about appearance. Nash et al. (2019) studied whether people with high levels of narcissism regulate their anguish through approval on social networks. Results indicate that validation on social networks reduced the anguish caused by social exclusion for those with a greater sense of leadership and mastery. Sion’s (2019) study on selfies posted by American adults on social media and Barry et al.’s (2019) research on the publication of selfies on Instagram and the self-perception of university students offer an analysis on the communication of emotions through visual content. Results indicate that users can post self-portraits as a way of acting in accordance with a prevailing cultural norm. Along these lines, Wang et al.’s (2017) research on the psychological effects of posting and viewing selfies and group posts on social networks emphasizes that recurring viewing of selfies may be linked to decreased satisfaction with life, in contrast to a group view associated with fuller satisfaction.

Nonetheless, there are studies that show that social networks, through influencers, can help create social awareness around different topics. Mañas-Viniegra et al. (2019) conducted a study to determine how attention is paid to fashion advertising and awareness-raising around physical appearance by curvy influencers compared to advertising by fashion brands on Instagram. They carried out a biometric eye tracking on a sample of 120 participants from Spain and Portugal, whose profile coincided with that of the main users of the social network under study: urban women under 25, interested in fashion and who perceived themselves as curvy. The results indicate these curvilinear influencers are raising awareness, focusing more on imperfections than on the same fashion items that they promote.

### Emotions: User Reactions, Elements That Determine Them and Engagement Implications

When analyzing the object of study from the user’s perspective and reviewing the existing bibliography that addresses this issue, there are three thematic trends that stand out above the rest: the analysis and categorization of possible emotional reactions by users, the attempts to identify the possible elements that influence these reactions, and the implications that users’ emotional reactions may have in terms of engagement.

Regarding the first of these two questions, Brynielsson et al. (2014) developed a tool to classify user reactions on Twitter during a crisis and identified four main categories that correspond to the same number of user reactions. Specifically, they refer to positive reactions, fear, anger, or other. In the first case, they collected the reactions that show happiness or, at least, positive feelings. In the second, they collected reactions that reveal that people are scared, worried or afraid for some reason. In the third, they collected the reactions of users who showed anger or disappointment. Finally, the category of “others” is defined by exclusion, and groups all those reactions that do not correspond to any of the other three categories indicated.

Different authors delve into some of these specific feelings or emotions. Some of them relate the consumption of content on Facebook to the appearance of positive emotions (Mauri et al., 2011; Lin and Utz, 2015). Thus, for example, a feeling of well-being, coupled with a highly positive valence and a high level of excitement are usually the most common reactions (Mauri et al., 2011).

In turn, Lin and Utz (2015) delved into these aspects and analyzed the influence of the strength of the existing link on the reaction that arises in a person who reads a post on Facebook. These authors referred to two main mechanisms to explain this phenomenon, one of which is closely related to emotions: emotional contagion and upward social comparison. Both are closely connected with two of the most common emotional responses in these cases and which, as different authors have shown, can be given in online communication and not only face-to-face: happiness (Cheshin et al., 2011; Coviello et al., 2014) and envy (Tandoc et al., 2014).

The feelings manifested by the subjects who participated in the study by Lin and Utz (2015) are mostly positive. Specifically, they define themselves as connected, informed or entertained. On the other hand, when it comes to negative feelings –much less common– subjects are defined as envious, jealous, annoyed, and frustrated. The final conclusions of these authors suggest that when users browse Facebook, positive emotions prevail over negative ones. The second conclusion has important implications for brands: the strength of the existing link facilitates the generation of a feeling of happiness or, in the case of envy, it is benign. Meanwhile, when the link does not exist or is not as strong, malicious envy is more likely to appear, even if the tone of the message that has been read on Facebook is positive.

Stieglitz and Dang-Xuan (2013), researching political communication on Twitter in Germany, insisted on this idea, concluding that messages that contain some kind of emotional power (regardless of whether it is positive, negative, or mixed) are much more likely to be viralized in some way (shared, retweeted, etc.) than those that don’t. The reason is that these are messages that have a much greater probability of generating some type of reaction in the user and thus make them feel the need to share them, incorporating their own personal point of view on the subject. Chmiel et al. (2011) offered a complementary element to this vision: the emergence of some kind of emotion and, beyond that, of collective emotional states –that is, of emotions shared by the different people who feel that way– is the key element to the creation and permanence of online user communities over time.

Meanwhile, Min and Yun (2019) focused on anger and determined, in the field of political communication and in South Korea, that this emotion plays a fundamental role in social networks when it comes to promoting or intensifying the force of social mobilizations. These authors concluded that the emergence of negative emotions, especially anger, is a more determining factor when the number of participants in a social protest increases, much more than the specific object of that protest or other factors, such as the personal agenda or the greater or lesser availability of the participants.

From a more pure business perspective, trying to analyze the relationship between the company and customers in social media, Sashi (2012) defined four basic profiles in terms of emotional ties and relational exchanges: the transactional customer (a profile characterized by the low connection both in the emotional bonds that are established and in the relational exchanges that develop), the delighted customer (high emotional bond and low relational exchange), the loyal customer (low emotional bond and high relational exchange), and fans (both emotional ties and relational exchanges are high). All of this, in turn, has interesting applications in terms of engagement between the company and customers, which this author specifies in what he calls the engagement cycle, consisting of seven stages: connection, interaction, satisfaction, retention, commitment, advocacy, and engagement. According to these conclusions, knowing these four profiles and their reactions, the company’s strategy should be based on identifying its audiences and, above all, detecting the presence of fans, from which that emotional link will be created.

Coviello et al. (2014) delved into the analysis of positive emotions, especially happiness. Their study implies that, on the one hand, emotional contagion also works online, through social networks. In fact, the magnitude of this contagion is intensified. And, on the other hand, it can reach different parts of the world, that is, it can reach subjects who initially had not interacted with the protagonists of the beginning of that process. In this sense, these authors insist on the need to be cautious when extrapolating their incidence in smaller and more specific cities or geographical nuclei.

Considering the elements that are studied as possible influential agents in the generation of these reactions, Sano et al. (2019) analyzed the influence of the temporal element on the emergence of these collective emotions. This study is significant because it collected user reactions on social media in Japan throughout over 10 years (2006–2016). The conclusion is that there are specific periods that are repeated year after year and in which specific emotional states are generated. They are periods associated, on the one hand, with special dates, such as Christmas Eve and Day, New Year’s Eve, the beginning of the holidays, Thanksgiving Day or Valentine’s Day and, on the other hand, to specific but relevant events, especially catastrophes or natural phenomena that alter the development of daily life (such as earthquakes, typhoons, heavy snow, among others). Although the authors warned that their results would need a greater basis than only the reactions of users in social media, this study still suggests a possible future line of study.

Another of the trends detected in the literature is the analysis of socio-cultural differences, addressed by several authors (Hudson et al., 2016; Lee and Hong, 2016; Ng and Kozlowski, 2018). In the first case, the authors conducted a study on the relations between brands and their users developed in the United States, the United Kingdom, and France, concluding that contextual differences may constitute one of the elements that decisively influence emotional reactions on the users. Ng and Kozlowski (2018) developed a similar analysis in Australia and Singapore, and concluded that there is a positive correlation between the development of positive emotional reactions and the feeling of well-being. At the same time, these authors detected that, on the contrary, there is no such relationship between the intensity of activity in social activities and the feeling of well-being. Lee and Hong (2016) approached these questions from the perspective of attitudinal beliefs and social influences, and concluded that the first element is more important in the user’s reaction than the second: the perception of what the rest think (the environment, society in general, etc.) influences, but not as much as previous beliefs, the development of empathy with the brand.

On the other hand, several authors (Teixeira et al., 2012; Nelson-Field et al., 2013; Lewinski et al., 2014) focused on analyzing the effects of the videos included in messages when generating these reactions. The most significant thing about these studies is that the emergence of reactions, especially positive ones, such as happiness, joy or even surprise, influence other elements such recalling the video, the way in which it is retained and, consequently, the opinion that the user has about that message. However, Schreiner et al. (2019), reviewing the literature that addresses these and other questions, concluded that more research is needed on this subject in order to reach results that can be considered more reliable.

Finally, another of the localized trends is the relationship between emotions, user reactions and engagement. Thus, the main conclusion reached by studies dealing with these issues is that the emotions developed by users also significantly influence the level of engagement that can be generated among those who publish messages on social networks (brands, politicians, private users, etc.) and the public that receives these messages (Hollebeek and Chen, 2014; Ferrara and Yang, 2015; Hudson et al., 2015; Marbach et al., 2016; Dessart, 2017; Fan et al., 2018). In fact, Ferrara and Yang (2015) studied the transmission of emotions through Twitter, identifying two types of users: highly and scarcely susceptible to emotional contagion. These authors also determined that the former are much less predisposed than the latter to develop negative emotions. However, regarding positive emotions, no significant differences between each other were detected. Therefore, in general, the probability of developing positive feelings is greater.

Hudson et al. (2015) studied the emotional connection from the perspective of music festivals, with implications also for engagement between the organizers and the public. They developed a scale composed of ten categories: affectionate, friendly, loved, peaceful, passionate, delightful, captivated, connected, bored, and attached. The results show that social networks contribute significantly to the generation of these emotions, all of them positive in one way or another, which in turn leads to desired results in terms of word of mouth and engagement.

Dessart (2017) draws important conclusions for brands. The author analyzed 48 Facebook pages, which corresponded to nine different product categories, and concluded that the degree of emotional involvement by users toward the communities that are regularly created around a brand is one of the elements that determines the level of engagement, not only with that community, but also with the brand itself, toward which trust, commitment, and loyalty can be generated. According to this author, for a user, the fact that a brand responds to a comment on social networks would have, in emotional terms, a similar value, and would generate an equally positive feeling, to have an interaction with other members of the community. In short, engagement with a community can be considered as a precursor to engagement with the brand.

Marbach et al. (2016) analyzed the influence of personality traits and emotions on the development of engagement between brands and users. Specifically, the traits that can play some kind of influence are seven: introversion/extroversion, (dis)agreeableness, conscientiousness, openness to experience, need for activity, need for learning and altruism. It is a classification with very important implications in terms of user segmentation by brands.

As can be seen, in general, the studies that address these aspects do so taking into account positive emotions. However, there are also authors who have analyzed the impact of negative emotions (Hollebeek and Chen, 2014; Fan et al., 2018). The conclusions of Hollebeek and Chen (2014) can be related to those previously mentioned by Dessart (2017), who suggest that when it comes to generating engagement related to negative emotions, the connection with the brand plays a more relevant role than that which can be established with a user community. Fan et al. (2018) find that negative emotions are more easily transmitted in the case of user networks whose connection is weak, while positive feelings are more likely to be channeled in those other networks in which the ties are closer and more consolidated. Furthermore, negative feelings, and specifically anger, are more likely to become dominant when some public event occurs that also has negative connotations (attack, murder, etc.).

## Discussion

The analysis of the research reviewed throughout this article allow to draw a series of conclusions that demonstrate the effectiveness of neuromarketing as a tool for studying the relationships between companies and consumers in social networks:

1. Neuromarketing has shown that advertising content that directly appeals to emotions, mainly sadness, obtain better levels of effectiveness than those that try to convey a purely rational message, regardless of the medium (Ambler et al., 2000; Baraybar-Fernández et al., 2017; Hafez, 2019; Harris et al., 2019).
2. Neuromarketing techniques indicate that the use of social networks occurs because users need to satisfy their social needs, to find validation and to make a good impression on their network, which encourages them to act largely based on the emotions and behaviors they observe in others (Meshi et al., 2013, 2015; Turel et al., 2018).
3. Engagement between companies and consumers improves on social networks when they post entertainment content. Business or corporate social responsibility posts do not drive user interaction (de Vries et al., 2012; Cvijikj and Michahelles, 2013; Khan et al., 2016).
4. In times of corporate crisis, users consider the reputation of organizations more positive when they read positive and defensive comments than when they only read the news (Hong and Cameron, 2017; Xu and Wu, 2017; Vignal Lambret and Barki, 2018), since they value the opinion of other users very much, more than that of the media. These data indicate that, during critical moments, organizations have to design strategies that generate engagement and encourage the most committed users and their stakeholders to come to their defense to preserve their reputation.
5. Messages on social networks that carry an emotional charge, both positive and negative, generate reactions on the users, so they present higher levels of virality (Hollebeek and Chen, 2014; Marbach et al., 2016; Fan et al., 2018).
6. Another important element to assess the relationship between organizations and consumers is that studies show that when they interact with the user on social networks, for example, responding to a comment, the sentiment generated is just as positive as when the individual interacts with other members of community (Dessart, 2017).
7. Facebook is by far the most analyzed social network in the most recent studies on neuromarketing applied to social networks. This social platform is the object of study in researches by Mauri et al. (2011), de Vries et al. (2012), Cvijikj and Michahelles (2013), Goh et al. (2013), Meshi et al. (2013), Nelson-Field et al. (2013), Coviello et al. (2014), Hollebeek and Chen (2014), Tandoc et al. (2014), Aguirre et al. (2015), Hudson et al. (2015), Kim et al. (2015), Lin and Utz (2015), Khan et al. (2016), Lee and Hong (2016), Marbach et al. (2016), Dessart (2017), Hwong et al. (2017), Kim and Yang (2017), Schultz (2017), Swani and Milne (2017), Swani et al. (2017), Wang et al. (2017), Turel et al. (2018), Vermeulen et al. (2018), Vignal Lambret and Barki (2018), Muñoz-Leiva et al. (2019), Sion (2019), and Smith and Seitz (2019).
8. Business communication and personal communication are the most studied subject areas in neuromarketing applied to social networks. Business communication is presented as the main subject of research by Chmiel et al. (2011), de Vries et al. (2012), Teixeira et al. (2012), Cvijikj and Michahelles (2013), Goh et al. (2013), Hollebeek and Chen (2014), Hudson et al. (2015), Hudson et al. (2016), Kim et al. (2015), Khan et al. (2016), Marbach et al. (2016), Dessart (2017), Kim and Yang (2017), Schultz (2017), Swani and Milne (2017), Swani et al. (2017), Xu and Wu (2017), Rout et al. (2018), Vignal Lambret and Barki (2018), and Moussa (2019). Personal communication is analyzed in studies by Mauri et al. (2011), Meshi et al. (2013), Nelson-Field et al. (2013), Brynielsson et al. (2014), Coviello et al. (2014), Tandoc et al. (2014), Carrillo et al. (2015), Ferrara and Yang (2015), Lin and Utz (2015), Lee and Hong (2016), Wang et al. (2017), Fan et al. (2018), Ng and Kozlowski (2018), Turel et al. (2018), Vermeulen et al. (2018), Barry et al. (2019), Min and Yun (2019), Nash et al. (2019), Ranganathan and Tzacheva (2019), and Sion (2019).

These conclusions show the usefulness of neuromarketing as a tool to improve communication between companies and users on social networks, given its ability to determine what type of messages work best and what type of multimedia content they prefer. With this data, companies can optimize their communication strategies, avoid crises, and protect their reputation on social networks.

Likewise, this study has allowed to detect a series of future trends whose research would contribute to deepening the study of social networks through neuromarketing. In this sense, the following needs have been detected:

1. Studies that delve into the influence that social networks are exerting on the purchase decision of certain products and services. In this sense, it would be necessary to analyze how reading positive or negative comments influences the purchase of a certain product or service.
2. Deepening studies that allow analyzing the impact of social networks on behavior change or awareness of certain social problems through influencers.
3. When analyzing the reactions of users, research could delve into the effect of negative appreciations, since until now studies that focus on positive ones have predominated.

## Author Contributions

NV worked in the introduction, research design, results, and discussion. JD-C and DR worked in the research design, results, and discussion. All authors contributed to the article and approved the submitted version.

## Conflict of Interest

The authors declare that the research was conducted in the absence of any commercial or financial relationships that could be construed as a potential conflict of interest.
